# Quiet Time: Gene Program Prevents Division but Keeps Cells at the Ready

**DOI:** 10.1371/journal.pbio.0040085

**Published:** 2006-03-07

**Authors:** Richard Robinson

Because uncontrolled cell division is so dangerous for an organism, the well-behaved cell must know not only when to divide, but also—crucially—when not to. Shutting down cell division prevents tumors and maintains the proper form of tissues such as muscle. Many cells, though, including fibroblasts, must also retain the ability to start dividing again when conditions are right—when the organism must grow, or a damaged tissue must be repaired. A cell in such a temporary, nondividing state is said to be “quiescent.” Signals that send a cell into quiescence include loss of contact with the underlying surface, too much contact with neighboring cells, and not receiving specific growth factors from the surroundings.

Despite its importance, little is known about the quiescent state. In a new study, Hilary Coller, Liyun Sang, and James Roberts define the genetic underpinnings of quiescence, showing that it is actively maintained by a host of genes. Different signals induce genetically different quiescence states, but all share a core set of genes that define a “quiescence program.” They also show that these gene changes distinguish quiescence from irreversible nondividing states, such as the terminal differentiation of a mature muscle.

The authors began by treating fibroblasts with one of the three quiescence signals, and used DNA microarrays to identify genes whose expression increased or decreased as a result of the treatment. Fourteen hours after treatment, the three signals had induced three distinct gene profiles, with only a handful of overlapping genes. The common genes included several powerful transcription factors, each of which regulate multiple other genes. Over time, however, the number of overlapping genes increased. After 20 days, there were over 100 genes whose change in expression linked them to quiescence. These included not only those that regulate metabolism and cell division, as might be expected, but also genes that suppress the transition to two other cell fates—differentiation and programmed death. The expression of these genes (along with many others) was increased, indicating the active nature of the quiescent state.

The reversibility of quiescence contrasts with the cell cycle arrest induced by inhibition of cyclin-dependent kinase (CDK), a key regulatory protein. When the authors treated fibroblasts with a CDK inhibitor, division stopped, but the quiescence program was not activated, and the cells could be induced to irreversibly transform into muscle precursor cells by treatment with the differentiation signal, MyoD. Quiescent cells, on the other hand, were resistant to MyoD-induced differentiation, in keeping with the reversible nature of quiescence.

The identification of different quiescent states, induced by the three different signals, may lead to a better understanding of context-specific control of cell growth during development and repair, not only in muscle, but perhaps in other tissues as well. Identification of specific genes that enforce quiescence may also lead to better strategies for controlling cell division, including the unchecked division of cancer.

